# Depression symptoms, HIV testing, linkage to ART, and viral suppression among women in a high HIV burden district in KwaZulu-Natal, South Africa: A cross-sectional household study

**DOI:** 10.1177/1359105320982042

**Published:** 2020-12-31

**Authors:** Kaymarlin Govender, Dick Durevall, Richard G Cowden, Sean Beckett, Ayesha BM Kharsany, Lara Lewis, Gavin George, Cherie Cawood, David Khanyile

**Affiliations:** 1 University of KwaZulu-Natal, South Africa; 2 University of Gothenburg, Sweden; 3 Harvard University, USA; 4 University of KwaZulu-Natal, South Africa; 5 Epicentre AIDS Risk Management (Pty) Limited, South Africa

**Keywords:** Mental health, HIV/AIDS, UNAIDS fast track, 90-90-90, Depression, HIV care cascade

## Abstract

Achieving the UNAIDS 90-90-90 targets by 2020 is contingent on identifying and addressing mental health challenges that may affect HIV testing and treatment-related behaviors. This study is based on survey data from KwaZulu-Natal, South Africa (2014–2015). HIV positive women who reported higher depression scores had a lower odds of having tested previously for HIV (15–25 years: AOR = 0.90, 95% CI [0.83, 0.98]; 26–49 years: AOR = 0.90, 95% CI [0.84, 0.96]). Because HIV testing behavior represents a gateway to treatment, the findings suggest mental health may be one challenge to attaining the UNAIDS 90-90-90 targets.

## Background

The 90-90-90 targets of 2020 have become central to worldwide efforts focused on achieving epidemic control (UNAIDS, 2017). These cascaded goals aim for 90% of people living with HIV to be diagnosed and know their status (first 90), 90% of those diagnosed to receive antiretroviral treatment (ART) (second 90), and 90% of those on ART to be virally suppressed (third 90) (UNAIDS, 2014). This strategy is expected to reduce the HIV epidemic to one new HIV incidence per 1000 population by 2030 (Jamieson and Kellerman, 2016), as 73% of all people living with HIV would be virally suppressed if the 90-90-90 targets are achieved (UNAIDS, 2014). Substantial progress has been made towards reaching these targets (Marsh et al., 2019), yet recent estimates indicate that only about half of the global population of people living with HIV are virally suppressed (UNAIDS, 2017). The challenge of attaining the 90-90-90 targets is amplified in HIV hyper-endemic contexts and where key populations are disproportionately affected by the disease (UNAIDS, 2014).

South Africa contains the largest number of people living with HIV worldwide (Closson et al., 2018). South Africa also has the highest rate (33%) of new HIV infections in Eastern and Southern Africa (UNAIDS, 2018), a region where global burden of the HIV epidemic is highest (Govender et al., 2018). HIV incidence rates in South Africa are higher among females aged 15 years and older compared to males of the same age (Simbayi et al., 2018). Recent estimates indicate that modest progress has been made towards the 90-90-90 targets in South Africa, and that progress is most notable for the third 90 (Grobler et al., 2017; Huerga et al., 2018).

Collective evidence suggests that global achievement of the 90-90-90 targets by 2020 is contingent on identifying and addressing key challenges to HIV testing, treatment uptake, and treatment adherence in settings where the HIV burden is highest (i.e., South Africa) and among subpopulations that are particularly vulnerable to HIV infection (i.e., young women). Although a range of barriers impeding attainment of the 90-90-90 targets have been identified (Granich et al., 2017), improved understanding of salient mental health conditions is needed to maximize intervention efforts aimed at the 90-90-90 goals.

Depression is the leading cause of disability worldwide (Friedrich, 2017). Approximately 4.4% of the global population are living with depression (World Health Organization, 2017). Prevalence estimates consistently identify higher rates of depression among women (Baxter et al., 2014; Whiteford et al., 2013; World Health Organization, 2017), increasing their risk of chronic health comorbidities that accompany depression (Moussavi et al., 2007; Smith et al., 2014). Specifically, depression is associated with high-risk sexual behaviors (e.g., early sexual debut, multiple sex partners) (Agardh et al., 2012; Logie et al., 2018b; Rubin et al., 2009) that are linked with HIV (Berg et al., 2007; Steffenson et al., 2011; Stöckl et al., 2013), indicating that depression may heighten susceptibility to HIV infection. Research on the mental health of individuals seeking HIV testing (i.e., HIV status unknown) indicates that depression may precede receiving notification of an HIV positive test result (Kagee et al., 2017). Severe depression is associated with late HIV testing and diagnosis among individuals who are HIV positive (Rane et al., 2018), resulting in delayed entry into care and treatment programs.

Conversely, there is widespread evidence supporting HIV as a risk factor for depression (Nanni et al., 2015; Pappin et al., 2012). Individuals who receive an HIV positive diagnosis often experience internalizing symptoms (e.g., shame, guilt, internalized stigma) that may trigger or exacerbate existing depression (Li et al., 2009; Rodkjaer et al., 2010). HIV seropositive individuals tend to score higher on measures of depression (Morrison et al., 2002), with rates of depression higher among HIV positive females compared to their male counterparts (Robertson et al., 2014). Depression is commonly linked to ART non-adherence among HIV infected individuals (Kidia et al., 2015), with recent findings highlighting depression as a self-reported explanation for non-adherence to viral suppression medication (Betancur et al., 2017). Research also suggests that chronic depressive symptoms are associated with higher viral loads and lower CD4 counts (Crawford and Thornton, 2017; Ickovics et al., 2001; Yehia et al., 2015), irrespective of medication adherence (Amanor-Boadu et al., 2016).

Few studies have been undertaken to understand links between depression, HIV testing, and linkage to ART as indicated in the 90-90-90 targets, particularly among subpopulations at higher risk of contracting HIV who reside within HIV hyper-endemic regions. Using a population-based sample of South African women, the current study intended to examine associations of depression symptoms with HIV testing and linkage to care variables indicated in three pillars of the HIV treatment cascade. It was hypothesized that women living with HIV who have higher depression symptoms would be less likely to have undergone HIV testing (and therefore less likely to know their status), less likely to be on HIV treatment, and less likely to have a suppressed virus load.

## Methods

### Participants and procedure

This study is a secondary analysis of the data from the HIV Incidence Provincial Surveillance System (HIPSS) project in two sub-districts (Vulindlela and Edendale) in uMgungundlovu District, KwaZulu-Natal province in South Africa (Kharsany et al., 2015). uMgungundlovu is a HIV hyper-endemic district with an antenatal HIV prevalence of 44% in 2016. Vulindlela is considered largely rural and Greater Edendale is largely peri-urban. Data were collected between June 2014 and June 2015. Households were randomly selected using a three-stage random sampling technique: (1) selection of enumerator areas, (2) selection of households (drawn randomly within enumeration areas), and (3) random selection of one eligible individual between 15 and 49 years of age from each household to participate in the study. Although 9812 participants were sampled in total, the current study focused exclusively on females who were HIV positive (*N* = 2955).^1^

Face-to-face interviews were conducted by trained fieldworkers. The survey battery consisted of socio-demographics, health-related measures, and HIV-related measures. Venous blood samples were collected from all participants and tested for HIV antibodies and antigens with the Biomérieux Vironostika Uniform II Antigen/Antibody Microelisa system (BioMérieux, Marcy I’Etoile, France) and HIV 1/2 Combi Roche Elecsys (Roche Diagnostics, Penzberg, Germany). Positive tests were confirmed with a HIV-1 Western-Blot assay (Biorad assay, Bio-Rad Laboratories, Redmond, WA 98052, USA). In HIV seropositive blood samples, HIV-1 Ribonucleic acid viral load measurements were established with Roche COBAS Ampliprep/COBAS TaqMan HIV-1 version 2.0 assay (TaqMan, Roche Diagnostics, Mannheim, Germany).

The HIPSS protocol was approved by the Center for Disease Control and Prevention (CDC) of the Center for Global Health (CGH 2014-080), the KwaZulu-Natal Provincial Department of Health in South Africa (HRKM 08/14), and the Biomedical Research Ethics Committee, University of KwaZulu-Natal (BF269/13). Eligible participants provided written informed consent. Legal minors provided assent and written consent was obtained from parents, guardians, or caregivers.

### Measures

#### Depression

The 10-item Center for Epidemiological Studies Depression Scale (CES-D10) was used to measure symptoms of depression (Kohout et al., 1993). The CES-D10 is a 10-item screening instrument that provides a continuous measure of depressive symptoms experienced in the past week (e.g., “I felt depressed”), two of which are reverse scored (range = 0–30). The factorial and cross-cultural validity of the CES-D10 has been supported in a variety of subpopulations and languages (Björgvinsson et al., 2013; Kilburn et al., 2018; Zhang et al., 2012), including those native to South Africa (Baron et al., 2017). Reported internal consistency estimates have been appropriate (⩾0.79) across a number of studies (Björgvinsson et al., 2013; Jang et al., 2009; Zhang et al., 2012). In the current sample, α = 0.80.

Participants completed two self-report items on HIV status (“Have you been tested to see if you are HIV positive?” and “What was the result of your latest HIV test?”) and receipt of sustained ART (“Are you still taking ARV’s?”) using a dichotomous (0 = *No*; 1 = *Yes*) response scale. Use of antiretroviral drugs lamivudine, emtricitabine, nevirapine, efavirenz, and lopinavir was measured by mass spectrometry using electrospray ionization-positive mode (QTRAP 6500+; AB SCIEX) in the plasma of a sample of HIV positive respondents to assess the accuracy of self-reported ART drug use. There was no evidence of inconsistencies in self-reported ART use in this sample. Those data are not reported herein, as only a small subsample of cases were checked by laboratory assistants. Participants who had viral load values of ⩽1000 copies per mL were classified as virally suppressed.

### Data analyses

All analyses were conducted using Stata 15. The analyses included weights to account for the probability of selecting the enumeration area, the household in the enumeration area, and the individual in the household, which then were adjusted for nonresponse and benchmarked to the size of the population in the study area (for details, see Grobler et al., 2017). A series of multiple logistic regression analyses were conducted to assess relations between depression symptoms, having tested for HIV, and the 90-90-90 treatment cascade. As the risk of HIV infection and the impact of depression symptoms might differ between young and middle-aged females, separate models were estimated for younger and older women (i.e., 15–25 years and 26–49 years, respectively).

Previous studies that have used the CES-D10 to screen for depression symptoms have typically taken one of two approaches to operationalizing depression. Some studies have used cut-off points on the CES-D10 (e.g., 8, 10, 12) to identify individuals at risk for depression (Andresen et al., 1994; Baron et al., 2017; Zhang et al., 2012). The alternative approach is consistent with a substantive body of evidence that indicates psychological problems are best viewed as continuous constructs rather than discreet categories (Siddaway et al., 2017; Wood et al., 2010). That is, researchers have opted to use the CES-D10 as a continuous index of depression symptoms (Logie et al., 2018a), including those that have been conducted in South Africa (Ardington and Case, 2010). To optimize analytical sensitivity in modeling outcomes associated with symptoms of depression, logistic regression models were estimated by operationalizing CES-D10 scores as a continuum of depression symptoms.^2^

As a first step, we examined the association between depression symptoms and the probability of ever having undergone a HIV test among those who tested HIV positive. The association of depression symptoms with each of the three components of the UNAIDS 90-90-90 targets were assessed in the following ways: The first 90, that 90% of the people living with HIV should know that they are HIV positive was assessed by estimating the association between depression symptoms and the probability that a HIV positive woman has knowledge of her HIV status. The second 90, having initiated treatment and the third 90, viral load suppression, were estimated using the sample of those who self-reported being HIV positive and had been advised to start treatment (the second component) and those who self-reported that they were taking ART (the third component). Statistical models were estimated (a) while controlling for age only and (b) while controlling for age, educational attainment, marital status, and whether the person spent more than a month away from home in the previous year. We included education (De Walque, 2009), marital status (Venkatesh et al., 2011) and time away from home (Tanser et al., 2015) as confounders in the regression models, as these variables have been shown to be associated with the use of HIV services.

### Data sharing statement

The complete ﻿dataset,﻿ syntax, and output for this study are available on 10.6084/m9.figshare.13378877.

## Results

Participant characteristics are reported in Table 1. A majority of the participants were between 26 and 49 years of age (77.5%), had not completed secondary school (56.2%), and were not married (82.6%). Almost all of the participants (98.9%) reported Zulu as their primary language. A majority of the sample (85.0%) had previously tested for HIV. Nearly three fourths (74.8%) of HIV positive participants knew their HIV positive status. Most participants (92.8%) who had been told they were eligible for HIV treatment indicated they were on treatment. Viral suppression was detected in 89.5% of the sample who indicated receiving ART.

**Table 1.** Demographic and HIV-related characteristics of HIPSS sample, uMgungundlovu 2014/2015.

	Age 15–25 (*n* = 663)	Age 26–49 (*n* = 2292)	Total (*N* = 2955)
Participant characteristics			
Age, *M* (SD)	21.83 (2.5)	36.00 (6.6)	32.82 (8.4)
Married or living together, *n* (%)	44 (6.6)	496 (20.5)	513 (17.4)
Completed secondary school or above, *n* (%)	334 (50.4)	959 (41.8)	1293 (43.8)
Primary language Zulu, *n* (%)	656 (99.3)	2255 (98.8)	2911 (98.9)
>1 month away from home in the past 12 months, *n* (%)	78 (11.1)	233 (10.2)	311 (10.6)
Depression symptoms, *M* (SD)	7.11 (4.4)	7.20 (4.5)	7.18 (4.5)
HIV testing and UNAIDS 90-90-90 targets			
HIV test, *n* (%)	532 (80.2)	1.981 (86.4)	2.513 (85.0)
Know HIV positive, *n* (%)	275 (58.5)	1558 (80.9)	1833 (74.8)
On antiretroviral treatment, *n* (%)	153 (87.4)	1098 (93.6)	1251 (92.8)
On antiretroviral treatment and suppressed viral load, *n* (%)	123 (80.4)	995 (90.8)	1118 (89.5)

Note. HIPSS, HIV incidence provincial surveillance system.

*M*: mean; *N*/*n*: sample size; SD: standard deviation.

Logistic regression model estimates are reported in Table 2. Both younger (Adjusted Odds Ratio (AOR) = 0.90, 95% Confidence Interval (CI) [0.83, 0.98], *p* < 0.05) and older women (AOR = 0.90, 95% CI [0.84, 0.96], *p* < 0.01) with higher depression scores were less likely to have previously tested for HIV. The regression for the first 90 did not reveal an association between depression scores and self-reported HIV positive status among younger or older women. For the second 90, depression scores were unrelated to the likelihood of being on ART in both age groups of HIV positive women. Results for the third 90 indicated that higher depression scores were associated with a reduced likelihood of being virally suppressed in younger women (AOR = 0.87, 95% CI [0.78, 0.97], *p* < 0.05), but not in older women.

**Table 2.** Logistic regression analysis for HIV testing and the UNAIDS 90-90-90 targets in the HIPSS study disaggregated by age, uMgungundlovu 2014/2015.

Predictor	Outcome			
	Living with HIV and have tested for HIV^ AOR [95%CI]	Living with HIV and status known AOR [95%CI]	Living with HIV and receiving ART AOR [95%CI]	Receiving ART and virally suppressed AOR [95%CI]
Females 15–25^#^				
Depression symptoms	0.91** [0.84, 0.99]	1.04 [0.97, 1.12]	0.94 [0.84, 1.05]	0.85** [0.75, 0.96]
*n*	663	524	153	153
Females 15–25^##^				
Depression symptoms	0.90** [0.83, 0.98]	1.04 [0.96, 1.12]	0.92 [0.81, 1.05]	0.86** [0.77, 0.96]
*n*	661	522	153	153
Females 26–49^#^				
Depression symptoms	0.89*** [0.84, 0.95]	0.98 [0.94, 1.02]	0.96 [0.89, 1.04]	1.01 [0.96, 1.06]
*n*	2292	1927	1175	1096
Females 26–49^##^				
Depression symptoms	0.90*** [0.85, 0.96]	0.97 [0.93, 1.01]	0.97 [0.90, 1.05]	1.00 [0.95, 1.06]
*n*	2283	1920	1172	1093

Note. ^All outcome variables are dichotomous (0 = *No*; 1 = *Yes*). ^#^Model controls for age only. ^##^Model controls for age, educational attainment, marital status, and whether the person spent >1 month away from home in the previous year. For full model details, see Supplemental File 1.

AOR: adjusted odds ratio; ART: antiretroviral treatment; CI: confidence interval; HIPSS: HIV incidence provincial surveillance system; *n*: sample size.

* *p* < 0.10. **p < 0.05. ***p < 0.01.

## Discussion

In this study, we examined relations between depression symptoms, HIV testing, and the UNAIDS fast track 90-90-90 cascade in a sample of women in a high HIV burden district within South Africa. Our findings indicate that women with higher depression symptoms were less likely to have previously tested for HIV, which aligns with recent evidence linking depression symptoms and non-utilization of HIV testing and counselling services among women (see Shrestha et al., 2017). Drawing on studies supporting relations of symptoms and diagnoses of depression with reduced mental health seeking behavior (Andersson et al., 2013; Umubyeyi et al., 2016), untreated symptoms of depression may have negative implications for HIV testing behavior and delay access to HIV treatment services. Removing barriers to seeking care from mental health professionals (e.g., perceived stigma) may increase engagement with mental health support, the indirect effects of which could be improved HIV testing and treatment-related behavior.

Results for the first 90 revealed that depression symptoms were unrelated to self-reported HIV status among women who are HIV positive. This finding might be a function of temporal changes in depressive symptoms that occur after learning of one’s HIV positive status. One study found that the initial increase in depressive symptoms following an HIV positive diagnosis tends to decline significantly to below the diagnostic threshold for possible depression after approximately 2 weeks, which suggests that knowing one’s HIV positive status may not be linked to ongoing depression symptoms (Kiene et al., 2018). Although we were unable to statistically control for length of time since HIV positive diagnosis in the subsample of women who were aware of their status based on the current data, research highlights the importance of ensuring appropriate mental health services are readily available and accessible to those who test positive for HIV (Remien et al., 2019).

For the second 90, there appears to be no association between depression symptoms and receiving ART. Considering a variety of factors (e.g., beliefs and attitudes towards ART, treatment-related knowledge, access to treatment facilities and quality of care) have been associated with late initiation of ART (for a review, see (Lahuerta et al., 2013), it may be that alternative processes have a more prominent influence on receipt of ART compared to the effects of depression symptoms. Using viral load data to assess the third 90, higher depression symptoms were associated with a reduced likelihood of being virally suppressed in younger women. The vulnerability of younger women to depression could be explained by lack of social support, given that only 6.6% of younger women were married or living with a partner. Retention in care, sustained ART, and viral suppression are unlikely to be achieved without treating depression, particularly among younger women.

The presence of depression symptoms has an influence on HIV testing behaviors. The findings of this study suggest that a lack of screening for depression among women living in a HIV hyper-endemic region may undermine HIV testing and the HIV treatment cascade. It would be beneficial to integrate depression screening into ongoing HIV care programs, which appears to be a crucial avenue for continuing progress towards realizing the UNAIDS 90-90-90 goals and ending the HIV epidemic. Given concerns over the cross-cultural validity of available measures of depression in sub-Saharan Africa, depression screening instruments ought to be locally adapted to ensure that the psychometrics of utilized measures are optimized for use in HIV and in non-HIV infected populations within this region (for reviews, see (Sweetland et al., 2014; Tsai, 2014)).

### Limitations

The findings of this study should be interpreted alongside select methodological limitations. First, depression was measured using a screening instrument (i.e., CES-D10) rather than via objective clinical evaluation performed by a qualified mental health professional. Second knowledge of HIV status, ART uptake, and depression symptoms were self-reported and susceptible to socially desirable response sets. Third, the cross-sectional data limits conclusions about the directionality and causality of relations between depression symptoms and the UNAIDS 90-90-90 cascade from being made. Fourth, the subsamples eligible for assessing the second and third components of the 90-90-90 cascade were small, particularly for younger women. Fifth, the findings are based on a sample of women drawn from a single high HIV burden district in a South eastern region of South Africa. Sixth, we were unable to reliably estimate the length of time since participants became aware of their HIV positive diagnosis. With some research indicating that the implication of knowing one’s HIV positive status for mental health is contingent on length of time since diagnosis (Kiene et al., 2018), it is possible that the link between depression symptoms and the first 90 is underestimated in the current data.

### Conclusion

This cross-sectional study examined the relations between depression, access to HIV testing, and the UNAIDS treatment cascades in a sample of women residing in an HIV hyper-endemic district in KwaZulu-Natal, South Africa. The findings of this study suggest that depression symptoms may be a critical barrier to HIV testing, which could delay attainment of the UNAIDS 90-90-90 targets. To improve HIV outcomes, there is a need to identify and remove barriers to seeking mental health care and improve access to mental health services, particularly for women who live in under-resourced, high HIV burden regions.

## Supplemental Material

sj-pdf-1-hpq-10.1177_1359105320982042 – Supplemental material for Depression symptoms, HIV testing, linkage to ART, and viral suppression among women in a high HIV burden district in KwaZulu-Natal, South Africa: A cross-sectional household study Click here for additional data file. Supplemental material, sj-pdf-1-hpq-10.1177_1359105320982042 for Depression symptoms, HIV testing, linkage to ART, and viral suppression among women in a high HIV burden district in KwaZulu-Natal, South Africa: A cross-sectional household study by Kaymarlin Govender, Dick Durevall, Richard G Cowden, Sean Beckett, Ayesha BM Kharsany, Lara Lewis, Gavin George, Cherie Cawood and David Khanyile in Journal of Health Psychology

sj-pdf-2-hpq-10.1177_1359105320982042 – Supplemental material for Depression symptoms, HIV testing, linkage to ART, and viral suppression among women in a high HIV burden district in KwaZulu-Natal, South Africa: A cross-sectional household study Click here for additional data file. Supplemental material, sj-pdf-2-hpq-10.1177_1359105320982042 for Depression symptoms, HIV testing, linkage to ART, and viral suppression among women in a high HIV burden district in KwaZulu-Natal, South Africa: A cross-sectional household study by Kaymarlin Govender, Dick Durevall, Richard G Cowden, Sean Beckett, Ayesha BM Kharsany, Lara Lewis, Gavin George, Cherie Cawood and David Khanyile in Journal of Health Psychology

## Notes

1 Men were excluded from the analysis because there was insufficient viral load and treatment data to reliably estimate effects for this subsample.

2 For readers who may be interested in supplementing the results that were estimated using continuous depression scores, Supplemental File 1 contains the results of an additional series of logistic regression analyses with the same HIV testing and linkage to care outcomes using two specific cut-off points (i.e., 8 and 12) for scores on the CES-D10. A value of 8 represents the lowest validated cut-off point reported in the literature on the CES-D10 (see Andersen et al., 1994), whereas a value of 12 is the highest cut-off point that has been recommended for use among Zulu-speaking South Africans (Baron et al., 2017).
