# Supplementary material for: Depression symptoms, HIV testing, linkage to ART, and viral suppression among women in a high HIV burden district in KwaZulu-Natal, South Africa: A cross-sectional household study
Source: J Health Psychol. 2020 Dec 31;27(4):936–45. doi: 10.1177/1359105320982042 (PMC8855385; doi:10.1177/1359105320982042)
Supplement: sj-pdf-1-hpq-10.1177_1359105320982042 – Supplemental material for Depression symptoms, HIV testing, linkage to ART, and viral suppression among women in a high HIV burden district in KwaZulu-Natal, South Africa: A cross-sectional household study [file sj-pdf-1-hpq-10.1177_1359105320982042.pdf]

Summary of Dataset and Analytic Procedure

The data for this study came from the HIV Incidence Provincial Surveillance System (HIPSS) project. Data collection for this project was conducted in two sub-districts (Vulindlela and Edendale) in uMgungundlovu District, KwaZulu-Natal province in South Africa. The data were collected between June 2014 and June 2015. Recruitment and data collection information are reported in Kharsany et al. (2015).

The analyses for this study were carried out with Stata 15. The data are stored in a Stata and an Excel file. The Stata code are included both below and in a Stata do file.

Most variables were recoded for the analyses. For example, *StillOnARV* has been recoded to *StillOnARVs_recode*, which is 0 if the participant is not on treatment and 1 if the participant is on treatment.

All available observations were included in each regression.

The number of valid cases is reported in Table 1, along with descriptive statistics (e.g., means, frequencies) for the study variables. Sample sizes vary across regressions because the proportion of participants that are eligible for each analysis varies by outcome. The number of valid cases for each regression analysis is indicated in Table 2, where the regression results are reported.

The variable *svyset* makes weighted adjustments based on the design of the survey. Details about how to account for weights and the design of the survey are provided in Grobler, Cawood, Khanyile, Puren, & Kharsany, 2017).

References

Grobler, A., Cawood, C., Khanyile, D., Puren, A., & Kharsany, A. B. (2017). Progress of UNAIDS 90-90-90 targets in a district in KwaZulu-Natal, South Africa, with high HIV burden, in the HIPSS study: a household-based complex multilevel community survey. *The Lancet HIV*, *4*, e505-e513.

Kharsany ABM, Cawood C, Khanyile D, et al. (2015) Strengthening HIV surveillance in the antiretroviral therapy era: rationale and design of a longitudinal study to monitor HIV prevalence and incidence in the uMgungundlovu District, KwaZulu-Natal, South Africa. *BMC public health* 15: 1149.

Stata code

```
use "C:\JHP_data.dta"

*Recoding, etc.

egen agedumtwo25 = cut(CurrentAge),at(15,18,20,22,24,26,50)
egen agedumtwo49 = cut(CurrentAge), at(15,26,28,30,32,34,36,38,40,42,44,46,48,50)
recode ARV (2=0) (1=1) , gen(ARV_rec_dum)
recode HIVTested (1=1) (2=0), gen (HIVTest_recode)
recode StillOnARV (1=1) (2=0), gen (StillOnARVs_recode)
recode viralload_number (0/1000=1) (1001/5400000 =0) , gen(Viralload_supp)
recode HIVStatus (1 = 1) (2=0) (3 = .)(98 = .) , gen(HIVStatus_recode)
recode marital_stat3 (1 = 1) (2 = 1) (3 = 0), gen(Married_Cohab)
recode AwayFromHome (1 = 1) (2=0) (98 = .) , gen(AwayFromHome_recode)

* Depression indicator, reversing the order, adding up the scores, and cut-offs
recode Happy (1=0) (2=1) (3=2) (4=3), gen(Happy_zero)
recode Hopeful (1=0) (2=1) (3=2) (4=3), gen(Hopeful_zero)

* Continous depression indicator Depressed_reversed
gen Depressed_reversed = Bothered_recode + Distracted_recode + Depressed_recode +
Effort_recode + fearful_recode ///
+ Restless_recode + Lonely_recode + Sluggish_recode + Happy_rec + Hopeful_rec
*Cut-offs
recode Depressed_reversed (0/7=0) (8/27=1) (98=.),gen (Depressed_all_8_C)
recode Depressed_reversed (0/11=0) (12/27=1) (98=.),gen (Depressed_all_12_C)


*Accounting for the design of the survey
svyset ea [pweight=genweight], fpc(pop) poststrata(age_gender) postweight(age_gen_total)
vce(linearized) || HHID, fpc(SelectHH) || _n, fpc(nrind)

*Regressions with continuous depression indicator, Depressed_reversed. Replace it with
Depressed_all_8_cut and Depressed_all_12_C to obtain results cut-off at 8 and 12.
HIV test
svy linearized, subpop(if Gender==2 & CurrentAge<=25 & hivresult==1): logit
HIVTest_recode Depressed_reversed i.agedumtwo25 , or
svy linearized, subpop(if Gender==2 & CurrentAge<=25 & hivresult==1): logit
HIVTest_recode Depressed_reversed  Educ2 Educ3 Educ4 Educ5 AwayFromHome_recode
Married_Cohab i.agedumtwo25 , or
svy linearized, subpop(if Gender==2 & CurrentAge>=26 & hivresult==1 ): logit
HIVTest_recode Depressed_reversed i.agedumtwo49 , or
svy linearized, subpop(if Gender==2 & CurrentAge>=26 & hivresult==1 ): logit
HIVTest_recode Depressed_reversed Educ2 Educ3 Educ4 Educ5 AwayFromHome_recode
Married_Cohab i.agedumtwo49 , or
```

```
* first 90, self reported HIV status, given HIV positive status
svy linearized, subpop(if Gender==2 & CurrentAge<=25 & hivresult==1): logit HIVStatus_r
Depressed_reversed i.agedumtwo25  , or
svy linearized, subpop(if Gender==2 & CurrentAge<=25 & hivresult==1): logit HIVStatus_r
Depressed_reversed  Educ2 Educ3 Educ4 Educ5 AwayFromHome_recode Married_Cohab
i.agedumtwo25 , or
svy linearized, subpop(if Gender==2 & CurrentAge>=26 & hivresult==1 ): logit HIVStatus_r
Depressed_reversed i.agedumtwo49 , or
svy linearized, subpop(if Gender==2 & CurrentAge>=26 & hivresult==1 ): logit HIVStatus_r
Depressed_reversed Educ2 Educ3 Educ4 Educ5 AwayFromHome_recode Married_Cohab
i.agedumtwo49 , or



*HIV StillOnARVs
svy linearized, subpop(if Gender==2 & CurrentAge<=25): logit StillOnARVs_recode
Depressed_reversed  i.agedumtwo25  , or
svy linearized, subpop(if Gender==2 & CurrentAge<=25 ): logit StillOnARVs_recode
Depressed_reversed Educ2 Educ3 Educ4 AwayFromHome_recode Married_Cohab
i.agedumtwo25 , or
svy linearized, subpop(if Gender==2 & CurrentAge>=26 ): logit StillOnARVs_recode
Depressed_reversed i.agedumtwo49 , or
svy linearized, subpop(if Gender==2 & CurrentAge>=26 ): logit StillOnARVs_recode
Depressed_reversed Educ2 Educ3 Educ4 Educ5 AwayFromHome_recode Married_Cohab
i.agedumtwo49 , or


*HIV Viralload_supp
svy linearized, subpop(if Gender==2 & CurrentAge<=25 & StillOnARVs_recode==1  ): logit
Viralload_supp Depressed_reversed  i.agedumtwo25 , or
svy linearized, subpop(if Gender==2 & CurrentAge<=25 & StillOnARVs_recode==1  ): logit
Viralload_supp Depressed_reversed Educ2 Educ3 Educ4 AwayFromHome_recode
Married_Cohab  i.agedumtwo25 , or
svy linearized, subpop(if Gender==2 & CurrentAge>=26 & StillOnARVs_recode==1 ): logit
Viralload_supp Depressed_reversed  i.agedumtwo49  , or
svy linearized, subpop(if Gender==2 & CurrentAge>=26 & StillOnARVs_recode==1 ): logit
Viralload_supp Depressed_reversed  Educ2 Educ3 Educ4 Educ5 AwayFromHome_recode
Married_Cohab i.agedumtwo49  , or
```
