# Supplementary material for: Depression symptoms, HIV testing, linkage to ART, and viral suppression among women in a high HIV burden district in KwaZulu-Natal, South Africa: A cross-sectional household study
Source: J Health Psychol. 2020 Dec 31;27(4):936–45. doi: 10.1177/1359105320982042 (PMC8855385; doi:10.1177/1359105320982042)
Supplement: sj-pdf-2-hpq-10.1177_1359105320982042 – Supplemental material for Depression symptoms, HIV testing, linkage to ART, and viral suppression among women in a high HIV burden district in KwaZulu-Natal, South Africa: A cross-sectional household study [file sj-pdf-2-hpq-10.1177_1359105320982042.pdf]

Supplemental File 1: Full model results for HIV testing and linkage to care outcomes regressed on symptoms of depression as a continuous variable and a dichotomized variable based on cut-off points of 8 and 12

The age-adjusted and full model results for relations between depression symptoms, HIV testing, and linkage to care reported in the manuscript are presented in Tables S1 to S4. Both age-adjusted and full model results for all outcomes based on cut-off points of 8 and 12 are reported in Tables S5 to S8 and Tables S9 to S12, respectively. Regardless of the approach used to operationalize depression, the results indicated that symptoms of depression are associated with a reduced odds of an HIV positive women previously testing for HIV. Moreover, the odds ratios for outcomes when depression was modeled as a continuous score were comparable to those obtained when cut-off points of 8 and 12 were used. For example, the adjusted odds ratio in the full model for HIV positive women having previously tested for HIV is 0.90 (see Table S1). This implies an odds ratio of 0.35 for a 10 unit increase in depression symptoms, which is comparable to adjusted odds of having previously tested for HIV among HIV positive women with self-reported depression symptoms ≥ 8 (i.e., 0.44) and ≥ 12 (i.e., 0.31) reported in Tables S5 and S9, respectively.

Regardless of whether depression symptoms were modeled as a continuous variable, dichotomously using a cut-off point of 8, or dichotomously using a cut-off point of 12, the results were similar for the first 90 among both younger and older groups of women (see Tables S2, S6, and S10). Results for the second and third 90s with depression symptoms modeled as a continuous variable were similar to those found for a cut-off point of 8 (see Tables S3, S4, S7, S8). In contrast, a cut-off point of 12 revealed a higher odds of receiving ART among younger women (see Table S11) and a higher odds of viral suppression among older women (see Table S12) when compared to corresponding models with depression symptoms specified as a continuous score (see Tables S3 and S4). However, the second and third 90s results in which depression symptoms were modeled using a cut-off point of 12 did not reach statistical significance. The differences among these results might be accounted for by the low quantities of participants that met the criteria for classification into the higher of the two depression symptom subgroups established using the predetermined cut-off points. That is, only 17.1% of women in the total sample reported depression symptoms ≥ 12 and 36.5% reported depression symptoms ≥ 8.

Table S1

Full model estimates for logistic regression analysis with depression symptoms modeled as a continuous variable regressed on HIV testing among HIV positive women in the HIPSS project, uMgungundlovu 2014/15

Model	Predictor	Outcome: Living with HIV and have tested for HIV AOR [95% CI]	
		Age 15 to 25 years	Age 26 to 49 years
Age-adjusted model	Depression symptoms	0.91** [0.84, 0.99]	0.89*** [0.84, 0.95]
	Age		
	15 to 17 years	(Reference)	-
	18 to 19 years	9.14*** [2.20, 37.91]	-
	20 to 21 years	23.93*** [7.88, 72.69]	-
	22 to 23 years	16.87*** [5.13, 55.49]	-
	24 to 25 years	21.99*** [6.95, 69.52]	-
	26 to 27 years	-	(Reference)
	28 to 29 years	-	2.68** [1.11, 6.45]
	30 to 31 years	-	2.36 [0.74, 7.50]
	32 to 33 years	-	2.04 [0.74, 5.62]
	34 to 35 years	-	3.74*** [1.47, 9.54]
	36 to 37 years	-	3.27** [1.30, 8.23]
	38 to 39 years	-	1.26 [0.46, 3.50]
	40 to 41 years	-	1.39 [0.58, 3.33]
	42 to 43 years	-	0.95 [0.33, 2.74]
	44 to 45 years	-	1.33 [0.49, 3.62]
	46 to 47 years	-	1.22 [0.38, 3.89]
	48 to 49 years	-	1.52 [0.63, 3.70]
	Constant	0.79 [0.21, 2.96]	12.26*** [6.17, 24.34]
	n	663	2,292
Full model	Depression symptoms	0.90** [0.83, 0.98]	0.90*** [0.85, 0.96]
	Age		
	15 to 17 years	(Reference)	-
	18 to 19 years	10.42*** [2.33, 46.67]	-
	20 to 21 years	31.23*** [8.76, 111.37]	-
	22 to 23 years	23.69*** [5.65, 99.39]	-
	24 to 25 years	31.51*** [8.19, 121.18]	-
	26 to 27 years	-	(Reference)
	28 to 29 years	-	2.72** [1.07, 6.93]
	30 to 31 years	-	2.20 [0.68, 7.15]
	32 to 33 years	-	2.02 [0.67, 6.13]
	34 to 35 years	-	3.65** [1.29, 10.32]
	36 to 37 years	-	3.39** [1.30, 8.86]
	38 to 39 years	-	1.31 [0.43, 3.93]
	40 to 41 years	-	1.34 [0.54, 3.32]
	42 to 43 years	-	0.87 [0.27, 2.74]
	44 to 45 years	-	1.45 [0.48, 4.40]
	46 to 47 years	-	1.22 [0.32, 4.70]
	48 to 49 years	-	2.19 [0.79, 6.10]
	Educational attainment		
	No school or incomplete primary schooling	(Reference)	(Reference)
	Primary schooling complete	4.93 [0.71, 34.46]	5.44*** [2.19, 13.50]
	Incomplete secondary schooling	7.70*** [1.91, 31.02]	5.99*** [3.36, 10.70]
	Completed secondary schooling	4.55** [1.10, 18.80]	5.71*** [2.90, 11.23]
	Tertiary	3.16 [0.49, 20.45]	6.38** [1.56, 26.15]
	Marital status		
	Unmarried	(Reference)	(Reference)
	Cohabiting or married	3.05 [0.41, 22.45]	1.11 [0.56, 2.17]
	Length of time away from home		
	Away from home ≤ 1 month in the past 12 months	(Reference)	(Reference)
	Away from home > 1 month in the past 12 months	1.76 [0.57, 5.49]	0.74 [0.35, 1.54]
	Constant	0.11** [0.01, 0.81]	2.21* [0.88, 5.57]
	n	661	2,283

Note. AOR, adjusted odds ratio; CI, Confidence interval; HIPSS, HIV Incidence Provincial Surveillance System; *n*, sample size. **p* < 0.10, ***p* < 0.05, ****p* < 0.01. Outcome variable is dichotomous (0 = *No*; 1 = *Yes*).

Table S2

Full model estimates for logistic regression analysis with depression symptoms modeled as a continuous variable regressed on the first UNAIDS 90-90-90 target among HIV positive women in the HIPSS project, uMgungundlovu 2014/15

Model	Predictor	Outcome: Living with HIV and status known AOR [95% CI]	
		Age 15 to 25 years	Age 26 to 49 years
Age-adjusted model	Depression symptoms	1.04 [0.97, 1.12]	0.98 [0.94, 1.02]
	Age		
	15 to 17 years	(Reference)	-
	18 to 19 years	0.60 [0.08, 4.49]	-
	20 to 21 years	0.64 [0.11, 3.62]	-
	22 to 23 years	1.34 [0.26, 6.92]	-
	24 to 25 years	2.30 [0.49, 10.77]	-
	26 to 27 years	-	(Reference)
	28 to 29 years	-	1.13 [0.56, 2.26]
	30 to 31 years	-	1.07 [0.48, 2.38]
	32 to 33 years	-	1.70 [0.79, 3.66]
	34 to 35 years	-	3.06*** [1.51, 6.21]
	36 to 37 years	-	1.64 [0.77, 3.48]
	38 to 39 years	-	2.26** [1.08, 4.75]
	40 to 41 years	-	1.78 [0.79, 4.01]
	42 to 43 years	-	1.34 [0.69, 2.63]
	44 to 45 years	-	2.26** [1.04, 4.90]
	46 to 47 years	-	1.70 [0.59, 4.93]
	48 to 49 years	-	1.56 [0.60, 4.08]
	Constant	0.63 [0.13, 3.05]	3.04*** [1.76, 5.26]
	n	524	1,927
Full model	Depression symptoms	1.04 [0.96, 1.12]	0.97 [0.93, 1.01]
	Age		
	15 to 17 years	(Reference)	-
	18 to 19 years	0.74 [0.10, 5.45]	-
	20 to 21 years	0.84 [0.15, 4.60]	-
	22 to 23 years	1.82 [0.36, 9.06]	-
	24 to 25 years	3.34 [0.75, 14.76]	-
	26 to 27 years	-	(Reference)
	28 to 29 years	-	1.10 [0.54, 2.23]
	30 to 31 years	-	1.18 [0.52, 2.71]
	32 to 33 years	-	1.54 [0.71, 3.37]
	34 to 35 years	-	2.90*** [1.42, 5.93]
	36 to 37 years	-	1.55 [0.71, 3.41]
	38 to 39 years	-	2.15* [0.96, 4.82]
	40 to 41 years	-	1.61 [0.67, 3.88]
	42 to 43 years	-	1.16 [0.57, 2.35]
	44 to 45 years	-	1.92 [0.83, 4.46]
	46 to 47 years	-	1.44 [0.54, 3.83]
	48 to 49 years	-	1.15 [0.44, 3.02]
	Educational attainment		
	No school or incomplete primary schooling	(Reference)	(Reference)
	Primary schooling complete	0.18 [0.01, 2.78]	0.24** [0.06, 0.97]
	Incomplete secondary schooling	0.08** [0.01, 0.70]	0.31* [0.09, 1.03]
	Completed secondary schooling	0.05*** [0.01, 0.44]	0.19*** [0.06, 0.62]
	Tertiary	0.06** [0.01, 0.59]	0.11*** [0.03, 0.42]
	Marital status		
	Unmarried	(Reference)	(Reference)
	Cohabiting or married	1.74 [0.61, 4.99]	0.99 [0.63, 1.56]
	Length of time away from home		
	Away from home ≤ 1 month in the past 12 months	(Reference)	(Reference)
	Away from home > 1 month in the past 12 months	1.03 [0.49, 2.16]	0.80 [0.41, 1.57]
	Constant	7.12 [0.44, 115.30]	14.75*** [3.74, 58.09]
	n	522	1,920

Note. AOR, adjusted odds ratio; CI, Confidence interval; HIPSS, HIV Incidence Provincial Surveillance System; *n*, sample size. *$p < 0.10$, **$p < 0.05$, ***$p < 0.01$. Outcome variable is dichotomous (0 = *No*; 1 = *Yes*).

Table S3

Full model estimates for logistic regression analysis with depression symptoms modeled as a continuous variable regressed on the second UNAIDS 90-90-90 target among HIV positive women in the HIPSS project, uMgungundlovu 2014/15

Model	Predictor	Outcome: Living with HIV and receiving ART AOR [95% CI]	
		Age 15 to 25 years	Age 26 to 49 years
Age-adjusted model	Depression symptoms	0.94 [0.84, 1.05]	0.96 [0.89, 1.04]
	Age		
	15 to 21 years	(Reference)	-
	22 to 23 years	1.09 [0.25, 4.77]	-
	24 to 25 years	0.73 [0.11, 4.94]	-
	26 to 27 years	-	(Reference)
	28 to 29 years	-	2.25 [0.34, 14.83]
	30 to 31 years	-	1.90 [0.43, 8.45]
	32 to 33 years	-	3.54* [0.82, 15.30]
	34 to 35 years	-	3.61 [0.75, 17.25]
	36 to 37 years	-	3.28 [0.79, 13.64]
	38 to 39 years	-	4.10 [0.73, 23.10]
	40 to 41 years	-	9.40*** [1.82, 48.67]
	42 to 43 years	-	2.11 [0.48, 9.30]
	44 to 45 years	-	10.51** [1.05, 105.41]
	46 to 47 years	-	4.18 [0.61, 28.43]
	48 to 49 years	-	5.17 [0.47, 56.78]
	Constant	13.13*** [3.62, 47.60]	7.98*** [1.88, 33.88]
	n	153	1,175
Full model	Depression symptoms	0.92 [0.81, 1.05]	0.97 [0.90, 1.05]
	Age		
	15 to 21 years	(Reference)	-
	22 to 23 years	1.03 [0.21, 4.99]	-
	24 to 25 years	0.57 [0.07, 4.50]	-
	26 to 27 years	-	(Reference)
	28 to 29 years	-	2.38 [0.42, 13.59]
	30 to 31 years	-	1.69 [0.40, 7.06]
	32 to 33 years	-	3.84* [0.91, 16.26]
	34 to 35 years	-	4.04* [0.84, 19.38]
	36 to 37 years	-	3.58* [0.82, 15.53]
	38 to 39 years	-	5.09* [0.88, 29.56]
	40 to 41 years	-	9.47*** [1.85, 48.53]
	42 to 43 years	-	2.25 [0.52, 9.73]
	44 to 45 years	-	12.47** [1.32, 117.48]
	46 to 47 years	-	6.04 [0.71, 51.65]
	48 to 49 years	-	6.19 [0.41, 93.30]
	Educational attainment		
	No school or incomplete primary schooling	(Reference)	(Reference)
	Primary schooling complete	0.03** [0.00, 0.92]	1.10 [0.19, 6.51]
	Incomplete secondary schooling	0.05** [0.00, 0.78]	1.75 [0.29, 10.39]
	Completed secondary schooling	0.05** [0.00, 0.62]	1.31 [0.21, 8.10]
	Tertiary	-	1.73 [0.18, 16.81]
	Marital status		
	Unmarried	(Reference)	(Reference)
	Cohabiting or married	59.85*** [5.33, 672.66]	0.60 [0.30, 1.20]
	Length of time away from home		
	Away from home ≤ 1 month in the past 12 months	(Reference)	(Reference)
	Away from home > 1 month in the past 12 months	2.62 [0.31, 22.04]	0.29*** [0.12, 0.69]
	Constant	274.99*** [16.76, 4511.79]	6.33* [0.78, 51.49]
	n	153	1,172

Note. AOR, adjusted odds ratio; ART, Antiretroviral treatment; CI, Confidence interval; HIPSS, HIV Incidence Provincial Surveillance System; *n*, sample size. $^*p < 0.10$, $^{**}p < 0.05$, $^{***}p < 0.01$. Outcome variable is dichotomous (0 = *No*; 1 = *Yes*).

Table S4

Full model estimates for logistic regression analysis with depression symptoms modeled as a continuous variable regressed on the third UNAIDS 90-90-90 target among HIV positive women in the HIPSS project, uMgungundlovu 2014/15

Model	Predictor	Outcome: Receiving ART and virally suppressed AOR [95% CI]	
		Age 15 to 25 years	Age 26 to 49 years
Age-adjusted model	Depression symptoms	0.85** [0.75, 0.96]	1.01 [0.96, 1.06]
	Age		
	15 to 17 years	(Reference)	-
	18 to 19 years	2.64 [0.14, 48.53]	-
	20 to 21 years	4.35 [0.33, 56.88]	-
	22 to 23 years	8.30* [0.80, 85.54]	-
	24 to 25 years	6.76* [0.73, 62.88]	-
	26 to 27 years	-	(Reference)
	28 to 29 years	-	5.48* [0.91, 32.94]
	30 to 31 years	-	0.52 [0.10, 2.65]
	32 to 33 years	-	1.07 [0.20, 5.69]
	34 to 35 years	-	0.66 [0.14, 3.20]
	36 to 37 years	-	1.04 [0.22, 4.89]
	38 to 39 years	-	1.29 [0.23, 7.23]
	40 to 41 years	-	1.32 [0.18, 9.74]
	42 to 43 years	-	2.13 [0.35, 12.92]
	44 to 45 years	-	3.05 [0.58, 16.19]
	46 to 47 years	-	1.02 [0.13, 7.73]
	48 to 49 years	-	1.44 [0.25, 8.11]
	Constant	2.72 [0.33, 22.41]	9.18*** [2.19, 38.44]
	n	153	1,096
Full model	Depression symptoms	0.86** [0.77, 0.96]	1.00 [0.95, 1.06]
	Age		
	15 to 17 years	(Reference)	-
	18 to 19 years	1.24 [0.09, 16.41]	-
	20 to 21 years	2.70 [0.35, 20.64]	-
	22 to 23 years	8.14** [1.34, 49.40]	-
	24 to 25 years	5.26* [0.85, 32.62]	-
	26 to 27 years	-	(Reference)
	28 to 29 years	-	6.30** [1.05, 37.88]
	30 to 31 years	-	0.70 [0.13, 3.91]
	32 to 33 years	-	1.48 [0.27, 8.03]
	34 to 35 years	-	0.82 [0.17, 3.99]
	36 to 37 years	-	1.37 [0.26, 7.25]
	38 to 39 years	-	2.07 [0.35, 12.33]
	40 to 41 years	-	1.95 [0.25, 15.21]
	42 to 43 years	-	3.21 [0.48, 21.45]
	44 to 45 years	-	4.41 [0.75, 25.97]
	46 to 47 years	-	1.97 [0.24, 16.25]
	48 to 49 years	-	2.52 [0.39, 16.41]
	Educational attainment		
	No school or incomplete primary schooling	(Reference)	(Reference)
	Primary schooling complete	0.39 [0.02, 7.64]	0.23 [0.02, 2.31]
	Incomplete secondary schooling	0.75 [0.05, 11.59]	0.24 [0.03, 2.14]
	Completed secondary schooling	0.26 [0.02, 4.50]	1.15 [0.11, 11.76]
	Tertiary	-	0.15 [0.01, 1.77]
	Marital status		
	Unmarried	(Reference)	(Reference)
	Cohabiting or married	1.51 [0.13, 17.11]	0.78 [0.37, 1.68]
	Length of time away from home		
	Away from home ≤ 1 month in the past 12 months	(Reference)	(Reference)
	Away from home > 1 month in the past 12 months	0.27* [0.06, 1.23]	0.50 [0.21, 1.21]
	Constant	8.37 [0.36, 196.24]	22.82** [1.84, 283.88]
	n	153	1,093

Note. AOR, adjusted odds ratio; ART, Antiretroviral treatment; CI, Confidence interval; HIPSS, HIV Incidence Provincial Surveillance System; *n*, sample size. *$p < 0.10$, **$p < 0.05$, ***$p < 0.01$. Outcome variable is dichotomous (0 = *No*; 1 = *Yes*).

Table S5

Full model estimates for logistic regression analysis with depression symptoms modeled as a dichotomous variable (cut-off point of 8) regressed on HIV testing among HIV positive women in the HIPSS project, uMgungundlovu 2014/15

		Outcome	
		Living with HIV and have tested for HIV AOR [95% CI]	
Model	Predictor	Age 15 to 25 years	Age 26 to 49 years
Age-adjusted model	Depression symptoms		
	< 8	(Reference)	(Reference)
	≥ 8	0.43** [0.19, 0.94]	0.38*** [0.23, 0.65]
	Age		
	15 to 17 years	(Reference)	-
	18 to 19 years	7.77*** [1.96, 30.84]	-
	20 to 21 years	21.71*** [7.04, 66.96]	-
	22 to 23 years	14.03*** [4.12, 47.76]	-
	24 to 25 years	20.16*** [6.17, 65.88]	-
	26 to 27 years	-	(Reference)
	28 to 29 years	-	2.66** [1.10, 6.42]
	30 to 31 years	-	2.22 [0.68, 7.23]
	32 to 33 years	-	1.89 [0.69, 5.17]
	34 to 35 years	-	3.48*** [1.36, 8.88]
	36 to 37 years	-	3.18** [1.28, 7.86]
	38 to 39 years	-	1.26 [0.45, 3.54]
	40 to 41 years	-	1.37 [0.57, 3.31]
	42 to 43 years	-	0.93 [0.33, 2.62]
	44 to 45 years	-	1.29 [0.48, 3.45]
	46 to 47 years	-	1.23 [0.39, 3.91]
	48 to 49 years	-	1.41 [0.59, 3.41]
	Constant	0.65 [0.21, 2.04]	7.91*** [4.18, 14.96]
	n	663	2,292
Full model	Depression symptoms		
	< 8	(Reference)	(Reference)
	≥ 8	0.44* [0.19, 1.03]	0.45*** [0.26, 0.77]
	Age		
	15 to 17 years	(Reference)	-
	18 to 19 years	8.48*** [2.02, 35.59]	-
	20 to 21 years	26.12*** [7.50, 90.97]	-
	22 to 23 years	18.36*** [4.66, 72.31]	-
	24 to 25 years	26.71*** [7.00, 101.84]	-
	26 to 27 years	-	(Reference)
	28 to 29 years	-	2.69** [1.06, 6.84]
	30 to 31 years	-	2.06 [0.62, 6.84]
	32 to 33 years	-	1.90 [0.64, 5.61]
	34 to 35 years	-	3.36** [1.20, 9.47]
	36 to 37 years	-	3.32** [1.29, 8.57]
	38 to 39 years	-	1.31 [0.44, 3.94]
	40 to 41 years	-	1.33 [0.53, 3.31]
	42 to 43 years	-	0.86 [0.28, 2.61]
	44 to 45 years	-	1.43 [0.48, 4.26]
	46 to 47 years	-	1.24 [0.32, 4.76]
	48 to 49 years	-	2.02 [0.73, 5.59]
	Educational attainment		
	No school or incomplete primary schooling	(Reference)	(Reference)
	Primary schooling complete	5.33* [0.76, 37.34]	5.40*** [2.20, 13.24]
	Incomplete secondary schooling	7.01*** [1.63, 30.18]	5.59*** [3.16, 9.91]
	Completed secondary schooling	4.40* [0.96, 20.28]	5.57*** [2.96, 10.46]
	Tertiary	2.99 [0.42, 21.11]	6.65*** [1.66, 26.57]
	Marital status		
	Unmarried	(Reference)	(Reference)
	Cohabiting or married	3.29 [0.39, 27.92]	1.12 [0.58, 2.15]
	Length of time away from home		
	Away from home ≤ 1 month in the past 12 months	(Reference)	(Reference)
	Away from home > 1 month in the past 12 months	1.40 [0.52, 3.74]	0.74 [0.35, 1.54]
	Constant	0.10** [0.01, 0.65]	1.47 [0.66, 3.31]
	n	661	2,283

Note. AOR, adjusted odds ratio; CI, Confidence interval; HIPSS, HIV Incidence Provincial Surveillance System; *n*, sample size. **p* < 0.10, ***p* < 0.05, ****p* < 0.01. Outcome variable is dichotomous (0 = *No*; 1 = *Yes*).

Table S6

Full model estimates for logistic regression analysis with depression symptoms modeled as a dichotomous variable (cut-off point of 8) regressed on the first UNAIDS 90-90-90 target among HIV positive women in the HIPSS project, uMgungundlovu 2014/15

Model	Predictor	Outcome	
		Living with HIV and status known AOR [95% CI]	
		Age 15 to 25 years	Age 26 to 49 years
Age-adjusted model	Depression symptoms		
	< 8	(Reference)	(Reference)
	≥ 8	1.44 [0.79, 2.63]	0.77 [0.49, 1.21]
	Age		
	15 to 17 years	(Reference)	-
	18 to 19 years	0.64 [0.09, 4.82]	-
	20 to 21 years	0.66 [0.12, 3.73]	-
	22 to 23 years	1.41 [0.28, 7.23]	-
	24 to 25 years	2.37 [0.51, 11.00]	-
	26 to 27 years	-	(Reference)
	28 to 29 years	-	1.13 [0.56, 2.27]
	30 to 31 years	-	1.07 [0.48, 2.39]
	32 to 33 years	-	1.68 [0.78, 3.63]
	34 to 35 years	-	3.06*** [1.51, 6.20]
	36 to 37 years	-	1.64 [0.77, 3.48]
	38 to 39 years	-	2.26** [1.08, 4.74]
	40 to 41 years	-	1.78 [0.79, 4.01]
	42 to 43 years	-	1.34 [0.68, 2.64]
	44 to 45 years	-	2.25** [1.03, 4.92]
	46 to 47 years	-	1.71 [0.58, 5.04]
	48 to 49 years	-	1.57 [0.60, 4.09]
	Constant	0.71 [0.16, 3.31]	2.86*** [1.75, 4.68]
	n	524	1,927
Full model	Depression symptoms		
	< 8	(Reference)	(Reference)
	≥ 8	1.29 [0.68, 2.42]	0.73 [0.47, 1.15]
	Age		
	15 to 17 years	(Reference)	-
	18 to 19 years	0.79 [0.11, 5.81]	-
	20 to 21 years	0.87 [0.16, 4.75]	-
	22 to 23 years	1.93 [0.39, 9.52]	-
	24 to 25 years	3.44 [0.79, 15.01]	-
	26 to 27 years	-	(Reference)
	28 to 29 years	-	1.10 [0.54, 2.25]
	30 to 31 years	-	1.18 [0.52, 2.71]
	32 to 33 years	-	1.52 [0.69, 3.35]
	34 to 35 years	-	2.89*** [1.42, 5.91]
	36 to 37 years	-	1.55 [0.71, 3.39]
	38 to 39 years	-	2.17* [0.97, 4.84]
	40 to 41 years	-	1.61 [0.67, 3.89]
	42 to 43 years	-	1.16 [0.58, 2.35]
	44 to 45 years	-	1.92 [0.82, 4.48]
	46 to 47 years	-	1.46 [0.54, 3.95]
	48 to 49 years	-	1.16 [0.45, 3.01]
	Educational attainment		
	No school or incomplete primary schooling	(Reference)	(Reference)
	Primary schooling complete	0.17 [0.01, 2.63]	0.24** [0.06, 0.92]
	Incomplete secondary schooling	0.08** [0.01, 0.75]	0.30** [0.09, 0.97]
	Completed secondary schooling	0.05*** [0.01, 0.47]	0.18*** [0.06, 0.60]
	Tertiary	0.06** [0.01, 0.65]	0.10*** [0.03, 0.40]
	Marital status		
	Unmarried	(Reference)	(Reference)
	Cohabiting or married	1.77 [0.63, 5.01]	1.00 [0.64, 1.57]
	Length of time away from home		
	Away from home ≤ 1 month in the past 12 months	(Reference)	(Reference)
	Away from home > 1 month in the past 12 months	1.11 [0.53, 2.34]	0.79 [0.40, 1.54]
	Constant	7.61 [0.49, 118.72]	13.70*** [3.74, 50.14]
	n	522	1,920

Note. AOR, adjusted odds ratio; CI, Confidence interval; HIPSS, HIV Incidence Provincial Surveillance System; *n*, sample size. *$p < 0.10$, **$p < 0.05$, ***$p < 0.01$. Outcome variable is dichotomous (0 = *No*; 1 = *Yes*).

Table S7

Full model estimates for logistic regression analysis with depression symptoms modeled as a dichotomous variable (cut-off point of 8) regressed on the second UNAIDS 90-90-90 target among HIV positive women in the HIPSS project, uMgungundlovu 2014/15

Model	Predictor	Outcome<br>Living with HIV and receiving ART<br>AOR [95% CI]<br>Age 15 to 25 years	 <br><br>Age 26 to 49 years
Age-adjusted model	Depression symptoms		
	< 8	(Reference)	(Reference)
	≥ 8	0.45 [0.11, 1.91]	0.55 [0.22, 1.34]
	Age		
	15 to 21 years	(Reference)	-
	22 to 23 years	1.09 [0.26, 4.61]	-
	24 to 25 years	0.72 [0.11, 4.74]	-
	26 to 27 years	-	(Reference)
	28 to 29 years	-	2.19 [0.32, 14.92]
	30 to 31 years	-	1.89 [0.42, 8.56]
	32 to 33 years	-	3.28 [0.69, 15.60]
	34 to 35 years	-	3.54 [0.71, 17.56]
	36 to 37 years	-	3.23 [0.75, 13.87]
	38 to 39 years	-	3.85 [0.63, 23.49]
	40 to 41 years	-	9.53*** [1.80, 50.51]
	42 to 43 years	-	2.06 [0.45, 9.49]
	44 to 45 years	-	10.50** [1.05, 104.53]
	46 to 47 years	-	4.29 [0.62, 29.66]
	48 to 49 years	-	5.03[0.45, 56.70]
	Constant	11.78*** [3.50, 39.60]	7.79*** [1.78, 34.13]
	n	153	1,175
Full model	Depression symptoms		
	< 8	(Reference)	(Reference)
	≥ 8	0.39 [0.08, 1.83]	0.61 [0.26, 1.42]
	Age		
	15 to 21 years	(Reference)	-
	22 to 23 years	0.96 [0.20, 4.62]	-
	24 to 25 years	0.58 [0.08, 4.29]	-
	26 to 27 years	-	(Reference)
	28 to 29 years	-	2.32 [0.40, 13.60]
	30 to 31 years	-	1.71 [0.41, 7.16]
	32 to 33 years	-	3.61* [0.78, 16.66]
	34 to 35 years	-	3.94* [0.79, 19.70]
	36 to 37 years	-	3.48 [0.78, 15.51]
	38 to 39 years	-	4.80* [0.77, 29.75]
	40 to 41 years	-	9.49*** [1.81, 49.61]
	42 to 43 years	-	2.19 [0.49, 9.83]
	44 to 45 years	-	12.36** [1.31, 116.38]
	46 to 47 years	-	6.41* [0.70, 58.40]
	48 to 49 years	-	5.95 [0.40, 88.78]
	Educational attainment		
	No school or incomplete primary schooling	(Reference)	(Reference)
	Primary schooling complete	0.03** [0.00, 0.92]	1.05 [0.18, 6.04]
	Incomplete secondary schooling	0.04** [0.00, 0.62]	1.58 [0.28, 8.79]
	Completed secondary schooling	0.05** [0.00, 0.56]	1.21 [0.21, 7.07]
	Tertiary	-	1.62 [0.17, 15.05]
	Marital status		
	Unmarried	(Reference)	(Reference)
	Cohabiting or married	52.52*** [5.01, 550.76]	0.59 [0.29, 1.21]
	Length of time away from home		
	Away from home ≤ 1 month in the past 12 months	(Reference)	(Reference)
	Away from home > 1 month in the past 12 months	2.14 [0.29, 15.87]	0.30*** [0.12, 0.71]
	Constant	270.05*** [16.19, 4503.49]	6.93* [0.91, 52.65]
	n	153	1,172

Note. AOR, adjusted odds ratio; ART, Antiretroviral treatment; CI, Confidence interval; HIPSS, HIV Incidence Provincial Surveillance System; *n*, sample size. $^*p < 0.10$, $^{**}p < 0.05$, $^{***}p < 0.01$. Outcome variable is dichotomous (0 = *No*; 1 = *Yes*).

Table S8

Full model estimates for logistic regression analysis with depression symptoms modeled as a dichotomous variable (cut-off point of 8) regressed on the third UNAIDS 90-90-90 target among HIV positive women in the HIPSS project, uMgungundlovu 2014/15

Model	Predictor	Outcome: Receiving ART and virally suppressed AOR [95% CI]	
		Age 15 to 25 years	Age 26 to 49 years
Age-adjusted model	Depression symptoms		
	< 8	(Reference)	(Reference)
	≥ 8	0.31* [0.09, 1.08]	1.07 [0.48, 2.43]
	Age		
	15 to 17 years	(Reference)	-
	18 to 19 years	1.63 [0.07, 36.18]	-
	20 to 21 years	2.78 [0.19, 40.47]	-
	22 to 23 years	4.95 [0.39, 63.68]	-
	24 to 25 years	4.90 [0.45, 54.02]	-
	26 to 27 years	-	(Reference)
	28 to 29 years	-	5.49* [0.91, 33.28]
	30 to 31 years	-	0.52 [0.10, 2.67]
	32 to 33 years	-	1.08 [0.20, 5.81]
	34 to 35 years	-	0.66 [0.14, 3.21]
	36 to 37 years	-	1.04 [0.22, 4.92]
	38 to 39 years	-	1.29 [0.23, 7.33]
	40 to 41 years	-	1.32 [0.18, 9.73]
	42 to 43 years	-	2.13 [0.35, 13.12]
	44 to 45 years	-	3.06 [0.57, 16.33]
	46 to 47 years	-	1.01 [0.13, 7.80]
	48 to 49 years	-	1.44 [0.25, 8.27]
	Constant	1.89 [0.18, 19.67]	9.40*** [2.32, 38.13]
	n	153	1,096
Full model	Depression symptoms		
	< 8	(Reference)	(Reference)
	≥ 8	0.32* [0.09, 1.22]	0.97 [0.46, 2.06]
	Age		
	15 to 17 years	(Reference)	-
	18 to 19 years	0.95 [0.06, 14.89]	-
	20 to 21 years	2.23 [0.27, 18.29]	-
	22 to 23 years	6.10* [0.90, 41.52]	-
	24 to 25 years	4.85 [0.70, 33.72]	-
	26 to 27 years	-	(Reference)
	28 to 29 years	-	6.26** [1.03, 37.97]
	30 to 31 years	-	0.70 [0.13, 3.94]
	32 to 33 years	-	1.47 [0.27, 8.11]
	34 to 35 years	-	0.82 [0.17, 4.03]
	36 to 37 years	-	1.36 [0.26, 7.30]
	38 to 39 years	-	2.06 [0.34, 12.45]
	40 to 41 years	-	1.95 [0.25, 15.22]
	42 to 43 years	-	3.22 [0.48, 21.78]
	44 to 45 years	-	4.42 [0.75, 26.18]
	46 to 47 years	-	1.97 [0.24, 15.97]
	48 to 49 years	-	2.52 [0.38, 16.65]
	Educational attainment		
	No school or incomplete primary schooling	(Reference)	(Reference)
	Primary schooling complete	0.50 [0.02, 11.55]	0.23 [0.02, 2.24]
	Incomplete secondary schooling	0.56 [0.03, 9.99]	0.24 [0.03, 2.12]
	Completed secondary schooling	0.21 [0.01, 4.08]	1.13 [0.11, 12.05]
	Tertiary	-	0.14 [0.01, 1.73]
	Marital status		
	Unmarried	(Reference)	(Reference)
	Cohabiting or married	1.60 [0.17, 15.20]	0.78 [0.36, 1.67]
	Length of time away from home		
	Away from home ≤ 1 month in the past 12 months	(Reference)	(Reference)
	Away from home > 1 month in the past 12 months	0.19* [0.04, 0.92]	0.50 [0.21, 1.21]
	Constant	6.90 [0.23, 207.31]	23.90** [1.90, 300.59]
	n	153	1,093

Note. AOR, adjusted odds ratio; ART, Antiretroviral treatment; CI, Confidence interval; HIPSS, HIV Incidence Provincial Surveillance System; *n*, sample size. *$p < 0.10$, **$p < 0.05$, ***$p < 0.01$. Outcome variable is dichotomous (0 = *No*; 1 = *Yes*).

Table S9

Full model estimates for logistic regression analysis with depression symptoms modeled as a dichotomous variable (cut-off point of 12) regressed on HIV testing among HIV positive women in the HIPSS project, uMgungundlovu 2014/15

Model	Predictor	Outcome<br>Living with HIV and have tested for HIV<br>AOR [95% CI]<br>Age 15 to 25 years	 <br><br>Age 26 to 49 years
Age-adjusted model	Depression symptoms		
	< 10	(Reference)	(Reference)
	≥ 10	0.31** [0.12, 0.81]	0.27*** [0.15, 0.50]
	Age		
	15 to 17 years	(Reference)	-
	18 to 19 years	8.23*** [2.00, 33.90]	-
	20 to 21 years	22.74*** [7.39, 69.95]	-
	22 to 23 years	16.01*** [4.87, 52.56]	-
	24 to 25 years	19.98*** [6.39, 62.47]	-
	26 to 27 years	-	(Reference)
	28 to 29 years	-	2.74** [1.12, 6.69]
	30 to 31 years	-	2.31 [0.72, 7.40]
	32 to 33 years	-	2.09 [0.76, 5.72]
	34 to 35 years	-	3.98*** [1.52, 10.38]
	36 to 37 years	-	3.32** [1.29, 8.50]
	38 to 39 years	-	1.29 [0.46, 3.64]
	40 to 41 years	-	1.39 [0.58, 3.31]
	42 to 43 years	-	0.91 [0.32, 2.56]
	44 to 45 years	-	1.36 [0.49, 3.73]
	46 to 47 years	-	1.26 [0.39, 4.02]
	48 to 49 years	-	1.51 [0.62, 3.72]
	Constant	0.55 [0.19, 1.64]	7.05*** [3.51, 14.13]
	n	663	2,292
Full model	Depression symptoms		
	< 10	(Reference)	(Reference)
	≥ 10	0.28** [0.10, 0.75]	0.31*** [0.17, 0.57]
	Age		
	15 to 17 years	(Reference)	-
	18 to 19 years	9.18*** [2.16, 38.98]	-
	20 to 21 years	29.36*** [8.25, 104.40]	-
	22 to 23 years	22.05*** [5.51, 88.26]	-
	24 to 25 years	28.85*** [7.66, 108.74]	-
	26 to 27 years	-	(Reference)
	28 to 29 years	-	2.82** [1.09, 7.25]
	30 to 31 years	-	2.16 [0.66, 7.09]
	32 to 33 years	-	2.06 [0.68, 6.23]
	34 to 35 years	-	3.87** [1.34, 11.19]
	36 to 37 years	-	3.30** [1.23, 8.85]
	38 to 39 years	-	1.32 [0.43, 4.00]
	40 to 41 years	-	1.32 [0.54, 3.24]
	42 to 43 years	-	0.83 [0.27, 2.53]
	44 to 45 years	-	1.47 [0.48, 4.50]
	46 to 47 years	-	1.23 [0.32, 4.72]
	48 to 49 years	-	2.16 [0.75, 6.19]
	Educational attainment		
	No school or incomplete primary schooling	(Reference)	(Reference)
	Primary schooling complete	5.35* [0.80, 35.81]	5.41*** [2.19, 13.34]
	Incomplete secondary schooling	8.45*** [2.20, 32.44]	5.74*** [3.29, 10.03]
	Completed secondary schooling	5.01** [1.36, 18.47]	5.53*** [2.87, 10.65]
	Tertiary	3.33 [0.53, 20.89]	6.45*** [1.61, 25.88]
	Marital status		
	Unmarried	(Reference)	(Reference)
	Cohabiting or married	2.58 [0.41, 16.15]	1.17 [0.61, 2.24]
	Length of time away from home		
	Away from home ≤ 1 month in the past 12 months	(Reference)	(Reference)
	Away from home > 1 month in the past 12 months	1.75 [0.53, 5.75]	0.70 [0.34, 1.45]
	Constant	0.07*** [0.01, 0.40]	1.35 [0.55, 3.31]
	n	661	2,283

Note. AOR, adjusted odds ratio; CI, Confidence interval; HIPSS, HIV Incidence Provincial Surveillance System; *n*, sample size. **p* < 0.10, ***p* < 0.05, ****p* < 0.01. Outcome variable is dichotomous (0 = *No*; 1 = *Yes*).

Table S10

Full model estimates for logistic regression analysis with depression symptoms modeled as a dichotomous variable (cut-off point of 12) regressed on the first UNAIDS 90-90-90 target among HIV positive women in the HIPSS project, uMgungundlovu 2014/15

Model	Predictor	Outcome: Living with HIV and status known AOR [95% CI]	
		Age 15 to 25 years	Age 26 to 49 years
Age-adjusted model	Depression symptoms		
	< 10	(Reference)	(Reference)
	≥ 10	1.03 [0.47, 2.27]	0.86 [0.46, 1.62]
	Age		
	15 to 17 years	(Reference)	-
	18 to 19 years	0.68 [0.09, 5.06]	-
	20 to 21 years	0.70 [0.13, 3.90]	-
	22 to 23 years	1.52 [0.30, 7.72]	-
	24 to 25 years	2.50 [0.54, 11.58]	-
	26 to 27 years	-	(Reference)
	28 to 29 years	-	1.13 [0.57, 2.26]
	30 to 31 years	-	1.06 [0.48, 2.35]
	32 to 33 years	-	1.71 [0.79, 3.68]
	34 to 35 years	-	3.05*** [1.50, 6.20]
	36 to 37 years	-	1.64 [0.77, 3.48]
	38 to 39 years	-	2.27** [1.08, 4.77]
	40 to 41 years	-	1.78 [0.79, 4.00]
	42 to 43 years	-	1.33 [0.68, 2.60]
	44 to 45 years	-	2.26** [1.04, 4.88]
	46 to 47 years	-	1.69 [0.58, 4.90]
	48 to 49 years	-	1.56 [0.60, 4.02]
	Constant	0.74 [0.16, 3.44]	2.71*** [1.69, 4.32]
	n	524	1,927
Full model	Depression symptoms		
	< 10	(Reference)	(Reference)
	≥ 10	0.95 [0.40, 2.26]	0.79 [0.42, 1.47]
	Age		
	15 to 17 years	(Reference)	-
	18 to 19 years	0.82 [0.11, 6.11]	-
	20 to 21 years	0.92 [0.17, 4.95]	-
	22 to 23 years	2.06 [0.42, 10.15]	-
	24 to 25 years	3.62* [0.83, 15.87]	-
	26 to 27 years	-	(Reference)
	28 to 29 years	-	1.11 [0.55, 2.24]
	30 to 31 years	-	1.16 [0.51, 2.66]
	32 to 33 years	-	1.55 [0.71, 3.40]
	34 to 35 years	-	2.89*** [1.40, 5.97]
	36 to 37 years	-	1.56 [0.71, 3.41]
	38 to 39 years	-	2.17* [0.97, 4.87]
	40 to 41 years	-	1.60 [0.66, 3.87]
	42 to 43 years	-	1.15 [0.56, 2.33]
	44 to 45 years	-	1.92 [0.83, 4.45]
	46 to 47 years	-	1.43 [0.53, 3.82]
	48 to 49 years	-	1.15 [0.44, 2.97]
	Educational attainment		
	No school or incomplete primary schooling	(Reference)	(Reference)
	Primary schooling complete	0.15 [0.01, 2.01]	0.25* [0.06, 1.04]
	Incomplete secondary schooling	0.07** [0.01, 0.63]	0.32* [0.09, 1.09]
	Completed secondary schooling	0.04*** [0.00, 0.38]	0.19*** [0.06, 0.65]
	Tertiary	0.05** [0.01, 0.50]	0.11*** [0.03, 0.45]
	Marital status		
	Unmarried	(Reference)	(Reference)
	Cohabiting or married	1.81 [0.65, 5.08]	1.01 [0.64, 1.58]
	Length of time away from home		
	Away from home ≤ 1 month in the past 12 months	(Reference)	(Reference)
	Away from home > 1 month in the past 12 months	1.20 [0.60, 2.40]	0.78 [0.40, 1.52]
	Constant	9.09 [0.60, 137.84]	12.21*** [3.24, 46.07]
	n	522	1,920

Note. AOR, adjusted odds ratio; CI, Confidence interval; HIPSS, HIV Incidence Provincial Surveillance System; *n*, sample size. *$p < 0.10$, **$p < 0.05$, ***$p < 0.01$. Outcome variable is dichotomous (0 = *No*; 1 = *Yes*).

Table S11

Full model estimates for logistic regression analysis with depression symptoms modeled as a dichotomous variable (cut-off point of 12) regressed on the second UNAIDS 90-90-90 target among HIV positive women in the HIPSS project, uMgungundlovu 2014/15

Model	Predictor	Outcome	
		Living with HIV and receiving ART AOR [95% CI]	
		Age 15 to 25 years	Age 26 to 49 years
Age-adjusted model	Depression symptoms		
	< 10	(Reference)	(Reference)
	≥ 10	2.27 [0.25, 20.78]	0.80 [0.26, 2.42]
	Age		
	15 to 21 years	(Reference)	-
	22 to 23 years	1.04 [0.22, 4.91]	-
	24 to 25 years	0.89 [0.13, 6.24]	-
	26 to 27 years	-	(Reference)
	28 to 29 years	-	2.33 [0.36, 15.20]
	30 to 31 years	-	1.86 [0.42, 8.20]
	32 to 33 years	-	3.68* [0.86, 15.75]
	34 to 35 years	-	3.66 [0.78, 17.23]
	36 to 37 years	-	3.32* [0.82, 13.46]
	38 to 39 years	-	4.25 [0.75, 24.10]
	40 to 41 years	-	9.47*** [1.84, 48.64]
	42 to 43 years	-	2.15 [0.49, 9.39]
	44 to 45 years	-	10.48** [1.05, 104.80]
	46 to 47 years	-	4.26 [0.63, 28.92]
	48 to 49 years	-	5.15 [0.48, 55.78]
	Constant	7.60*** [2.47, 23.44]	6.44*** [1.80, 23.07]
	n	153	1,175
Full model	Depression symptoms		
	< 10	(Reference)	(Reference)
	≥ 10	2.34 [0.18, 29.65]	0.87 [0.31, 2.48]
	Age		
	15 to 21 years	(Reference)	-
	22 to 23 years	0.99 [0.15, 6.48]	-
	24 to 25 years	0.77 [0.09, 7.03]	-
	26 to 27 years	-	(Reference)
	28 to 29 years	-	2.43 [0.43, 13.58]
	30 to 31 years	-	1.66 [0.40, 6.93]
	32 to 33 years	-	3.95* [0.94, 16.49]
	34 to 35 years	-	4.03* [0.85, 19.07]
	36 to 37 years	-	3.61* [0.85, 15.41]
	38 to 39 years	-	5.20* [0.90, 30.11]
	40 to 41 years	-	9.56*** [1.86, 49.17]
	42 to 43 years	-	2.27 [0.53, 9.74]
	44 to 45 years	-	12.59** [1.34, 118.56]
	46 to 47 years	-	6.14* [0.72, 52.73]
	48 to 49 years	-	6.21 [0.41, 94.81]
	Educational attainment		
	No school or incomplete primary schooling	(Reference)	(Reference)
	Primary schooling complete	0.04* [0.00, 1.54]	1.14 [0.20, 6.71]
	Incomplete secondary schooling	0.03** [0.00, 0.63]	1.83 [0.30, 11.15]
	Completed secondary schooling	0.04** [0.00, 0.54]	1.38 [0.22, 8.77]
	Tertiary	-	1.88 [0.18, 19.11]
	Marital status		
	Unmarried	(Reference)	(Reference)
	Cohabiting or married	59.24*** [4.91, 714.98]	0.61 [0.30, 1.22]
	Length of time away from home		
	Away from home ≤ 1 month in the past 12 months	(Reference)	(Reference)
	Away from home > 1 month in the past 12 months	1.57 [0.28, 8.88]	0.28*** [0.12, 0.69]
	Constant	192.04*** [8.10, 4551.72]	5.18 [0.71, 37.77]
	n	153	1,172

Note. AOR, adjusted odds ratio; ART, Antiretroviral treatment; CI, Confidence interval; HIPSS, HIV Incidence Provincial Surveillance System; *n*, sample size. **p* < 0.10, ***p* < 0.05, ****p* < 0.01. Outcome variable is dichotomous (0 = *No*; 1 = *Yes*).

Table S12

Full model estimates for logistic regression analysis with depression symptoms modeled as a dichotomous variable (cut-off point of 12) regressed on the third UNAIDS 90-90-90 target among HIV positive women in the HIPSS project, uMgungundlovu 2014/15

		Outcome	
		Receiving ART and virally suppressed AOR [95% CI]	
Model	Predictor	Age 15 to 25 years	Age 26 to 49 years
Age-adjusted model	Depression symptoms		
	< 10	(Reference)	(Reference)
	≥ 10	0.54 [0.08, 3.44]	2.59** [1.02, 6.57]
	Age		
	15 to 17 years	(Reference)	-
	18 to 19 years	1.17 [0.06, 21.89]	-
	20 to 21 years	1.88 [0.13, 27.24]	-
	22 to 23 years	3.43 [0.29, 41.13]	-
	24 to 25 years	3.64 [0.34, 38.61]	-
	26 to 27 years	-	(Reference)
	28 to 29 years	-	5.74* [0.96, 34.33]
	30 to 31 years	-	0.51 [0.10, 2.62]
	32 to 33 years	-	1.10 [0.21, 5.85]
	34 to 35 years	-	0.63 [0.13, 3.01]
	36 to 37 years	-	1.03 [0.22, 4.81]
	38 to 39 years	-	1.37 [0.25, 7.58]
	40 to 41 years	-	1.32 [0.18, 9.65]
	42 to 43 years	-	2.18 [0.36, 13.32]
	44 to 45 years	-	3.06 [0.58, 16.18]
	46 to 47 years	-	1.05 [0.14, 7.91]
	48 to 49 years	-	1.44 [0.26, 8.05]
	Constant	1.89 [0.18, 19.67]	8.78*** [2.31, 33.41]
	n	153	1,096
Full model	Depression symptoms		
	< 10	(Reference)	(Reference)
	≥ 10	1.03 [0.21, 5.02]	2.18 [0.84, 5.63]
	Age		
	15 to 17 years	(Reference)	-
	18 to 19 years	0.79 [0.06, 10.47]	-
	20 to 21 years	1.77 [0.19, 16.03]	-
	22 to 23 years	4.72 [0.66, 33.81]	-
	24 to 25 years	4.52 [0.65, 31.27]	-
	26 to 27 years	-	(Reference)
	28 to 29 years	-	6.41** [1.07, 38.47]
	30 to 31 years	-	0.66 [0.12, 3.71]
	32 to 33 years	-	1.46 [0.27, 7.89]
	34 to 35 years	-	0.77 [0.16, 3.73]
	36 to 37 years	-	1.33 [0.25, 7.07]
	38 to 39 years	-	2.08 [0.35, 12.25]
	40 to 41 years	-	1.91 [0.24, 14.83]
	42 to 43 years	-	3.10 [0.46, 20.71]
	44 to 45 years	-	4.34 [0.74, 25.67]
	46 to 47 years	-	1.97 [0.24, 16.26]
	48 to 49 years	-	2.39 [0.37, 15.49]
	Educational attainment		
	No school or incomplete primary schooling	(Reference)	(Reference)
	Primary schooling complete	0.94 [0.05, 17.09]	0.27 [0.03, 2.67]
	Incomplete secondary schooling	0.74 [0.05, 11.59]	0.28 [0.03, 2.49]
	Completed secondary schooling	0.25 [0.01, 4.12]	1.29 [0.13, 13.03]
	Tertiary	-	0.18 [0.02, 2.14]
	Marital status		
	Unmarried	(Reference)	(Reference)
	Cohabiting or married	1.57 [0.09, 26.51]	0.79 [0.37, 1.68]
	Length of time away from home		
	Away from home ≤ 1 month in the past 12 months	(Reference)	(Reference)
	Away from home > 1 month in the past 12 months	0.18 [0.04, 0.74]	0.50 [0.20, 1.24]
	Constant	4.49 [0.18, 112.36]	19.29** [1.60, 232.83]
	n	153	1,093

Note. AOR, adjusted odds ratio; ART, Antiretroviral treatment; CI, Confidence interval; HIPSS, HIV Incidence Provincial Surveillance System; *n*, sample size. **p* < 0.10, ***p* < 0.05, ****p* < 0.01. Outcome variable is dichotomous (0 = *No*; 1 = *Yes*).
